# Pulmonary Emphysema—Its Causes, Symptoms, and Treatment

**Published:** 1873-10

**Authors:** C. H. Tidd

**Affiliations:** Middleport, O.


### PULMONARY EMPHYSEMA—ITS CAUSES, SYMPTOMS, AND TREATMENT.

BY C. H. TIDD, M. D., OF MIDDLEPORT, O.

WRITTEN FOR THE MEIGS AND MASON ACADEMY OF MEDICINE, AND READ BEFORE THE SOCIETY THURSDAY EVENING, AUGUST 14, 1873.

By emphysema of the lung is meant either a morbid enlargement of the pulmonary vesicles, arising mainly from the blending of several vessels so as to form one great cyst—*emphysema versiculare*, (Niemeyer, vol. 1, p. 106), or else the escape of air into the subplural and interstitial connective tissue—"*emphysema interlobulare*." The opinion of authorities as to the origin and causes of emphysema, differ materially. We find four principal theories advanced upon the subject. According to one, it is caused by the immoderate or too protracted inflation of the air cells, by forced and long-continued inspiration. By another, it is said to be produced by forced expiration, causing excessive distension of the vesicular wall, According to a third author, its origin is not mechanical, but arises from nutritive derangement of the lung substance, which occurs independently of any strain or stretching. And lastly, a fourth claims that a morbid condition, namely, a rigid enlargement of the thorax, constitutes the primary disease, to which dilation of the air cells is only secondary.

Your essayist does not fully agree with any one of these views but believes that it can be proven that each of them is true in certain cases, while no single one of them will account for all forms of the disease. Different authors also have given different classifications to this disease, viz: interlobular, interstitial, subpleural, vesicular, vicarious, and substantive.

Such a vast array of names, we think, is only calculated to confuse the mind of the reader, and, in reality, there is no need of more than two, and in this paper we shall adhere to the classification of the older continental authors, which is vicarious and substantive. Vicarious representing those forms classed as interlobular and interstitial, may arise in one of two ways. First, in all cases in which portions of the lung substance become wasted, and shrink without corresponding reduction of the capacity of the thorax, by collapse of its wall. If the capacity of the chest remain constan f the size of the individual vesicles which contribute to fill it must, o course, depend upon their number; hence, if a portion of them perish, either a vacuum must be formed, or the remaining cells must dilate sufficiently to fill the cavity.

Secondly, it may arise when all of the vesicles do not participate alike in filling out the additional space found in the thorax by inspiratory dilatation. Normally, all the air-vesicles expand alike, by atmospheric pressure during inspiratory dilatation, but if some of them be filled with serum so that no more air can enter them, these cells will not dilate during inspiration; hence the remaining and healthy ones must expand for them, thus undergoing an abnormal degree of distension. As proof of this, we find in the bodies of all persons who have died of pneumonitis, those portions of healthy lung which remain to be emphysematous, and this condition seems to have been formed in the manner above stated—viz: vicariously.

Substantive emphysema may arise from immoderate and protracted inflation and stretching of the vesicular walls. But this form more commonly arises in the upper part of the lung as a result of oft repeated, forcible expiration with simultaneous contractions of the glottis, as in severe paroxysms in coughing, playing upon wind instruments, straining, lifting heavy weights, &c., the air being compressed within the thorax, and only allowed to escape at intervals. In such cases, contraction of the chest is affected by vigorous upheavals of the diaphragm. The result is, the expulsion of a strong current of air from the lower bronchi, the direction of which is obliquely upward, and if the air be prevented from escaping from or through the larynx, a portion of it must be driven into the upper bronchi, whose direction is obliquely downward. By the centrifugal force thus exerted by the compressed air upon the vesicles of the upper lobes of the lung, and upon the adjacent thoracic wall, the latter becomes distended as far as it is possible for them to yield, and emphysema results.

*Symptoms.*—Circumscribed vicarious emphysema, in the vicinity of portions of withered and shrunken lungs, can scarcely ever be recognized during life. The symptoms of vicarious and substantive emphysema are very similar to each other, and a differential diagnosis hard to make. Owing to the enlargement of the air cells, with destruction of many of the septa, a large number of the capillaries have also perished, and the breathing surface is very materially lessened. This fact, provided we have absence of other disease to account for the same, furnishes one strong point in making out a diagnosis.

In most cases we will find the sternum pushed decidedly forward, and the ribs also pushed upward and outward, and twisted upon their long axis; and as the costal cartilages are hypertrophied, and have become rigid, the thorax cannot return to its expiratory state, and we find the chest presenting constant signs of enlargement, and bulged outward, presenting a condition which has been very aptly termed a " rigid dilatation of the thorax."

When we find the costal cartilages bulging out over the entire chest, we can generally conclude that forced inspiration, with its attendant evils, is at the root of the difficulty; but if, on the contrary, we find the alteration confined to the upper ribs, we may conclude that forced expiration, with constriction of the glottis, was the primary seat of the trouble.

Another diagnostic point is, that parties who are suffering from the first-named cause cannot lie on their bellies or on either side. While those suffering from that caused by the second-named condition find infinite relief upon assuming either of those positions. Then again, the entire "facies" of the patient betrays obstruction of the respiration, dyspnea, oppression, and want of air, they summon all their force to open the thorax. The alæ nasi play, and the lower part of the neck becomes harder and broader from the energetic contraction of the scaline muscles with every inspiration. The patient will often pass the entire night in their chairs, fearing to choke if they lie down. The complexion becomes ashy and ruddy, the circulation slow and irregular, and the extremities cold; and in proportion as the circulation becomes embarrassed, we have another complication to combat—viz: hypertrophy of the right ventricle of the heart. The jugulars swell and throb painfully, the face becomes cyanatic, the lips swollen and blue, the cheeks assume a varicose appearance, and the patient complains of dizziness and headache, the liver becomes swollen and engorged from the overflow of blood being impeded, and the engorgement extends through the portal system to the gastric and intestinal veins, giving rise to gastric and intestinal catarrh. In the same manner the veins of the rectum become enlarged, causing blind piles, (*varices*).

In the latter stages of emphysema, the cyanosis becomes extremely intense. The cheeks, lips, ears and tongue of the patient are literally blue. In no other disease does this cyanosis become so intense, save in diseases of the orifices of the right heart, which are mostly congenital. All the other symptoms attributed to emphysema belong to its complications. Cough, for instance, points to a complication of chronic bronchitis; so to of many other symptoms which time will not permit us to enumerate. Percussion, also, forms an almost certain basis for diagnosis in emphysema; the sound being in most, but not all cases, unusually long and loud, but not tympanitic, and will be found to be abnormal in extent of the full clear sound of the lung, proving the statement that we made elsewhere, that in all cases of emphysema the diaphragm is abnormally depressed, and we find the sounds clear, as low down as the last true rib, instead of stopping at the sixth rib, as it normally does on the right side; and in emphysema of the left lung we find it extending to the 6th costal cartilage, instead of the 4th, and in severe cases the cardiac dullness entirely disappears. In the diagnosis we should carefully enquire also, as to whether the shortness of breath preceded the cough or came on after a severe attack of pertussis, and if the patient has been playing on wind instruments, and presents the characteristics above stated, we shall have at once a good starting point in our diagnosis.

In the treatment of emphysema, we can find no established and well beaten path. The physician is *obliged* to treat the complicatory symptoms, almost exclusively using hygienic measures, and he must feel, all through the course of treatment, that he is treating a disease which is beyond the skill of man to cure, and that, although the patient may carry an emphysematous lung for many years, that at last he will be forced to succumb to the ravages of disease through damage done to the parenchymatous substance of the lung. We would advise, though, the constant wearing of flannel next to the chest, the use of stimulating baths, vapors or warm baths, &c.

To obviate the habitual shortnesss of breath, the inhalation of preparations of belladonna, stramonnii, conii, &c., have been advised, and in some instances have afforded relief. Many times a removal during the summer months to a place where pine timber abounds, and where there is a heavy fall of dew, will be beneficial, the highly oxygenated air seeming to prove agreeable to patients. Latterly, the use of compressed air has been highly extolled, but experiments are wanting to prove its efficacy.

But after all these have been faithfully tried, our patient will usually fall into an early and premature grave, where, as Whittier justly says, "the sick and afflicted, poor and needy of this earth forget their trials and afflictions, and are happy in the perfect love of God."

